# Repeatability of baited remote underwater video station (BRUVS) results within and between seasons

**DOI:** 10.1371/journal.pone.0244154

**Published:** 2020-12-17

**Authors:** C. Samantha Sherman, Michelle R. Heupel, Mohini Johnson, Muslimin Kaimuddin, L. M. Sjamsul Qamar, Andrew Chin, Colin A. Simpfendorfer

**Affiliations:** 1 Centre for Sustainable Tropical Fisheries and Aquaculture; College of Science and Engineering, James Cook University, Townsville, Queensland, Australia; 2 AIMS@JCU, DB17-063, James Cook University, Townsville, Queensland, Australia; 3 Department of Biological Sciences, Earth to Oceans Research Group, Simon Fraser University, Burnaby, British Columbia, Canada; 4 Australian Institute of Marine Science, Cape Cleveland, Queensland, Australia; 5 Operation Wallacea, Spilsby, Lincolnshire, United Kingdom; 6 Wasage Divers, Wakatobi & Buton, Southeast Sulawesi, Indonesia; 7 Fisheries Department, Universitas Dayanu Ikhsanuddin, Bau Bau, Southeast Sulawesi, Indonesia; Institut de Recherche pour le Developpement, FRANCE

## Abstract

Baited remote underwater video stations (BRUVS) are increasingly being used to evaluate and monitor reef communities. Many BRUVS studies compare multiple sites sampled at single time points that may differ from the sampling time of another site. As BRUVS use grows in its application to provide data relevant to sustainable management, marine protected area success, and overall reef health, understanding repeatability of sampling results is vital. We examined the repeatability of BRUVS results for the elasmobranch community both within and between seasons and years, and explored environmental factors affecting abundances at two sites in Indonesia. On 956 BRUVS, 1139 elasmobranchs (69% rays, 31% sharks) were observed. We found consistent results in species composition and abundances within a season and across years. However, elasmobranch abundances were significantly higher in the wet season. The elasmobranch community was significantly different between the two sites sampled, one site being more coastal and easily accessed by fishermen. Our results demonstrate that while BRUVS are a reliable and repeatable method for surveying elasmobranchs, care must be taken in the timing of sampling between different regions to ensure that any differences observed are due to inherent differences amongst sampling areas as opposed to seasonal dissimilarities.

## Introduction

Sampling methods that yield consistent results under equivalent conditions are fundamental to ecological research [1, 2]. Completing research in a laboratory setting enables factors to be controlled for consistency to ensure results are a direct effect of what is being tested [3]. Additionally, laboratory experiments can be completed with a planned number of individuals to examine the consistency of results within a population [4, 5]. In a captive setting, however, animals may exhibit different behaviours [6], thus field experiments are necessary but must be carefully considered. In the field, researchers cannot control environmental conditions (e.g. water chemistry, light intensity, and other biota that may influence study species) [7]. Therefore, it is often difficult to have a completely controlled field study in a natural setting. Additionally, presenting novel equipment in an environment can also introduce sampling bias by increasing the likelihood of encountering higher risk-takers [8]. The use of reproducible methods can thus present a big challenge for researchers in ecological studies’ [9, 10]. Thus, there is a need to understand the consistency of a growing ecological sampling method.

Baited remote underwater video stations (BRUVS) are increasingly being used to survey predator abundances on coral reefs [11–13]. BRUVS have been shown to have higher statistical power and consistency than unbaited videos [14]. However, immediate resampling of an area has not yet been performed to determine repeatability of BRUVS surveys for elasmobranchs or other taxa. On a temporal scale, time of day has been shown to significantly affect the species observed on BRUVS [15, 16]. Only a single study has examined abundance of any species during different seasons and found season did not significantly affect presence of wedgefish (*Rhynchobatus* spp.) at BRUVS on the Great Barrier Reef [17]. Changes in rainfall between seasons can impact freshwater output, turbidity, and nutrient loads which may impact coastal species abundances including elasmobranchs [18, 19]. Many BRUVS studies compare locations that are sampled at a single time point [12, 20]. By only having a single sampling period, it is not possible to document if community composition and abundance estimates change seasonally. Such variation may present problems with interpretation of data when multiple sites that may have seasonally influenced residents are sampled during different seasons.

BRUVS sampling at different times of the year may result in different species being recorded as some may be migratory or seasonally resident. Seasonal movements have been documented in many marine animals with continental-scale seasonal migrations occurring in many species [21–23]. Smaller scale migrations have been observed in several elasmobranch species including manta rays (*Mobula* spp.) and eagle rays (*Aetobatus* spp.) [24–26]. There is little information on the movement patterns of tropical benthic rays and none related to seasonal migrations. In benthic rays, few studies have examined migratory behaviour and only in thornback rays (*Raja clavata*) has direct evidence of seasonal migration been noted [27, 28]. Other species, like blacktip reef sharks (*Carcharhinus melanopterus*) exhibit high site fidelity, with some individuals also capable of making longer range movements and use both coastal and offshore reef habitats throughout their lives [29, 30]. These movements do not appear to be seasonal, however, may be initiated by ontogenetic or other environmental factors [29].

Environmental factors are potential sources of variation in the abundance of species detected by BRUVS sampling. There are many environmental factors that can influence elasmobranch movement patterns and hence presence on BRUVS due to preferences for certain conditions [31]. Environmental factors can have varying levels of influence, with some being more important than others. These factors include: temperature [32, 33], salinity [34], phosphate levels [35], dissolved oxygen [3, 36], and tide [37, 38], among others. Acute disturbances such as tropical storms and floods can also temporarily alter local abundance and diversity of sharks [39]. In addition, environmental preferences are species-specific, meaning data is required for each species to determine likelihood of encountering a species in different conditions. Even in tropical coral reef ecosystems where climactic conditions remain relatively stable throughout the year there can be significant changes to the water characteristics seasonally [40].

Other influencing factors on elasmobranch presence at BRUVS may include bait and its associated parameters. For example, bait plume size, determined by currents, soak time, and initial bait weight can dramatically affect species and abundances observed on BRUVS [41]. Additionally, the type of bait used can also effect species present [42]. As coral reef monitoring using BRUVS grows in its capacity to provide data relevant to sustainable management, marine protected area success, and overall reef health, understanding repeatability of results is vital [11]. BRUVS are increasingly being used for sampling coral reef species diversity and abundance for a wide range of species. Therefore, understanding the repeatability of results from BRUVS sampling is vital for making conclusions from these studies. The aims of this paper are to: 1) determine repeatability of results from BRUVS sampling for elasmobranchs within and between years, and 2) determine seasonal differences in abundance, habitat use or assemblage of elasmobranchs during different seasons.

## Methods

### Study site

This research was carried out around Bau Bau, in Southeast Sulawesi on the island of Buton, Indonesia and has a fast-growing human population of over 150,000 [43]. Permits were issued through RISTEKDIKTI—permit number 32/SIP/FRP/E5/Dit.KI/I/2019. There are two distinct seasons through the year: wet and dry. The dry season begins in June lasting through November during which winds come from the southeast. The wet season begins in December and has prevailing winds from the west [44]. Despite having a wet and dry season, the area has heavy rainfall throughout the year with approximately 50 mm in the driest months and over 250 mm in the wettest months. Average air temperature is fairly consistent at approximately 25°C throughout the year. Average water temperature ranges from 24 to 32°C with colder temperatures recorded in August through October [45]. The sampling area near Bau Bau was split into two sites; one along the coast from the city centre to the southern tip of Buton (-5.68903 to -5.42543, 122.53928 to 122.62605, decimal degrees on WSG84 projection), and one consisting of three islands (Kadatua, Siompu, and Pulau Ular) each approximately 5 km from the main island of Buton (-5.64851 to -5.49524, 122.46853 to 122.55710, decimal degrees on WSG84 projection). Kadatua and Siompu each have a few small villages whose residents partake in subsistence fishing in adjacent waters using small vessels. Pulau Ular is uninhabited, however, many subsistence fishermen from the other islands and Bau Bau city fish around the island (Kaimuddin pers. obs). Similar to the rest of Indonesia, the primary animal protein consumed is fish, with it incorporated into at least 2 meals per day. This demand for fish protein has led to an extremely high level of both commercial and subsistence fishing that is underreported by up to 75% in Indonesia [46]. In particular, shark catch has been underreported and they are widely targeted due to the high value of their fins [47]. Stingrays are also frequently captured and retained and sold, regardless of size or worth [48].

### Sampling

A total of 956 successful BRUVS were deployed as per Sherman *et al*. (2018) at depths ranging from 1.5 m to 47.3 m, with an average depth of 19.7 ± 0.3 m. BRUVS were set for a minimum of one hour with an average deployment time of 75.5 ± 0.4 minutes. Up to six units were deployed simultaneously with at least 500 m between each unit. BRUVS consisted of aluminium frames that housed a GoPro Hero 4 Silver camera with wide angle view (approx. 170° in air), (1920 × 1080 video format, 30 frames/s) housed in NiMAR housings, and a bait arm that extended 1 m from the camera. The bait arm held a mesh bag containing approximately 1 kg of pilchards (Family: Clupeidae) or bonito (Family: Scombridae).

Sampling was repeated six times: late March 2017 and 2018 (wet season), July 2017 and 2018 (early dry season) and August 2017 and 2018 (late dry season). The two dry season sampling periods (labelled early and late for simplicity) enabled evaluation of the repeatability of results when abundance and species composition should be stable, making this the only way to determine repeatability. Sampling over two years allowed for analysis of repeatability between years. Finally, sampling in different seasons enabled evaluation of seasonal changes in elasmobranch abundances.

During both deployment and haul of BRUVS units, environmental factors recorded included: date, time, location (latitude/longitude), depth (m), cloud cover (%), tidal state (ebb, slack, flow), wind speed (Beaufort scale) and wind direction to account for environmental drivers of elasmobranch presence. Deployment times were split into three categories: morning (sets deployed from 7:00–10:29), midday (sets deployed from 10:30–13:29) and afternoon (sets deployed from 13:30–17:00) as some elasmobranchs have differing diel patterns [49, 50].

#### Species

Two species of shark were observed throughout this study: blacktip reef shark (*Carcharhinus melanopterus*) and whitetip reef shark (*Triaenodon obesus*). Identification to species level was not possible for all rays, specifically maskrays (genus: *Neotrygon*), eagle rays (genera: *Aetobatus* and *Aetomylaeus*), and devil/manta rays (genus: *Mobula*) making the exact number of species impossible to accurately estimate. At least 11 species of ray were observed, with up to 23 different species possibly observed throughout the study (S1 Table).

### Video analysis

BRUVS footage was analysed to record MaxN of all elasmobranch species using FinPrint Annotator (v.1.1.44.0). MaxN is the maximum number of individuals of a species observed in a single video frame. This was then converted to sightings per unit effort (SPUE) by dividing MaxN by the hours of video (MaxN/hr). Video footage was annotated by two independent annotators to minimise any individuals being missed, and species identification was validated by a senior reviewer. Visibility was assessed from video footage and categorised in two metre bins (0–2 m, 2.1–4 m… 10+ m) and then assigned the median value from the bin (i.e. 6.1–8 m bin would be assigned a value of 7). Visibility ranged from 1 to 10 m during each of the three seasons. Habitat and relief were determined by splitting the screen in a 5x4 square grid (20 squares total) using BenthoBox (www.benthobox.com). Each square within the grid that contained any benthos was assigned a relief score from 0 (flat) to 5 (complex) and the average score of all square containing relief was calculated. Reliefs with scores <1 indicate deployments in sandy habitats, whereas relief scores >2 indicate a deployment within the coral reef. Habitat was similarly assessed using the 20 squares. For each square, the dominant habitat category was selected and percent cover was calculated based on the total number of squares containing benthos. Possible benthos categories were hard coral, soft coral, bleached coral, unconsolidated (sand/rubble), consolidated (rock), seagrass, turf algae, macroalgae, sponge, true anemones, ascidians, crinoids, halimeda, hydrocoral, hydroids, and invertebrate complex (Holothurians, Echinoderms, molluscs, etc.).

### Statistical analysis

All statistics were performed using R (version 3.5.1) with standardised SPUE abundances for each species / species group unless otherwise stated. Due to the low sample size of larger rays that have similar ecological niches, all benthic stingrays with maximum disc widths over 1 m were combined for analyses and called “large stingrays”. A total of 95 rays in this category were observed from six species (*Himantura uarnak*, *Pateobatus fai*, *Pastinachus ater*, *Taeniurops meyeni*, *Urogymnus asperrimus* and *U*. *granulatus*). All sharks were also combined for analyses. This group was dominated by blacktip reef sharks (*Carcharhinus melanopterus*) and, therefore, they are the drivers of any patterns observed. The exact species identification of maskrays (Genus: *Neotrygon*) was not possible, therefore, all maskrays were combined for analysis. Similarly, eagle rays were often observed in the distance and it was not possible to determine species, thus all eagle rays were combined for analysis (Genera: *Aetobatus* and *Aetomylaeus*).

A PERMANOVA was run using the adonis2 function in the vegan package to determine differences in species composition between seasons, years, and sites. For this analysis, MaxN was used as it measures assemblage, not abundance. Similarly, the SIMPER following the PERMANOVA used MaxN The total MaxN of each species (except maskrays, eagle rays, and manta/devil rays, which were identified to genus) was calculated for 12 groups (every combination of season—wet, early dry, late dry; site—islands and coast; and year– 2017 and 2018). The SIMPER function in vegan was then used to determine the species contributing to differences in assemblage between levels of each significant factor identified in the PERMANOVA. A non-metric multi-dimensional scaling (nMDS) plot was created based on the resemblance values of the 12 groups with a minimum stress of 0.01 and 50 restarts.

ANOVAs were performed to determine any differences in SPUE of each species / species group between each season. Post-hoc Tukey tests were completed to determine where any significant differences occur. ANOVAs were used to demonstrate if relief and depth were significantly different between sites and seasons.

Generalised linear models (GLMs) were used (R package—glmmTMB [51]) to determine environmental factors driving species abundances. All six groups of elasmobranchs were analysed. Thirty-five ecologically relevant models plus a null model were run with SPUE of each elasmobranch group acting as the dependent variable. Models included the environmental variables recorded in the field such as time of day and wind speed, as well as shark presence (for models pertaining to ray presence only) (S2 Table for full list of models). The most parsimonious model within two Aikaike Information Criterion (AIC) units of the best performing model was selected [52, 53]. Variance inflation factors (VIF) were determined for all models to ensure there was no collinearity between variables [54]. Three distributions (negative binomial, zero-inflated negative binomial, and poisson) were tested for each species / species group and the best performing distribution, based on AIC and a Vuong test, was used for all models in that species / species group. Generalised boosted regression models (GBM) were performed in order to determine level of contribution of each factor included in selected models (R package—gbm [55]). GBMs were run with the inclusion of all BRUVS deployments, a tree complexity of 5, computer learning rate of 0.001, and a bag fraction of 0.5.

## Results

A minimum of 1139 elasmobranchs comprising 784 rays and 355 sharks were observed over 1202.45 hours of footage on 956 BRUVS (Table 1). Of the two shark species, blacktip reef sharks were far more abundant than whitetip reef sharks, making up 89.0% of all sharks observed. Maskrays comprised almost half of rays (47.1%), and ribbontail rays comprised a quarter (25.6%) of rays in this study. Eagle rays comprised 13.9% of rays observed. These three groups combined accounted for a large majority of rays observed (86.6%). Less than 10 individuals were observed from five different species and one genus of ray (Table 1).

**Table 1:** Abundances of elasmobranchs observed on BRUVS.

Common Name	Latin Name	Species Authority	Videos Present	Sum of MaxN
Bluespotted maskray complex	*Neotrygon* spp.	------------------------	250	369
Bluespotted ribbontail ray	*Taeniura lymma*	Forsskål, 1775	191	201
Eagle Rays	*Aetobatus / Aetomylaeus* spp.	------------------------	70	109
Coach whipray	*Himantura uarnak*	Gmelin, 1789	25	25
Pink whipray	*Pateobatis fai*	Jordan and Seale, 1906	32	57
Cowtail ray	*Pastinachus ater*	Annandale, 1909	7	7
Mangrove whipray	*Urogymnus granulatus*	Macleay, 1883	1	1
Porcupine whipray	*Urogymnus asperrimus*	Bloch and Schneider, 1801	2	2
Blotched fantail	*Taeniurops meyeni*	Müller and Henle, 1841	2	3
Bowmouth guitarfish	*Rhina ancylostoma*	Bloch and Schneider, 1801	1	1
Devil / Manta Ray	*Mobula* spp.	------------------------	3	3
Unknown Rays	----------------------	------------------------	5	6
Blacktip reef shark	*Carcharhinus melanopterus*	Quoy and Gaimard, 1824	272	316
Whitetip reef shark	*Triaenodon obesus*	Müller and Henle, 1837	38	38
Unknown Shark	----------------------	------------------------	2	2

Species and abundances of elasmobranchs observed on BRUVS in Bau Bau, Southeast Sulawesi, Indonesia.

### Elasmobranch assemblage

The PERMANOVA indicated a significant difference in elasmobranch assemblage between the two sites (coast and islands) (SS = 0.552, R^2^ = 16.423, p <0.001). No significant differences in elasmobranch assemblage were observed between seasons or years (season: SS = 0.109, R^2^ = 0.174, p = 0.075; year: SS = 0.024, R^2^ = 0.038, p = 0.298).

**Fig 1:**
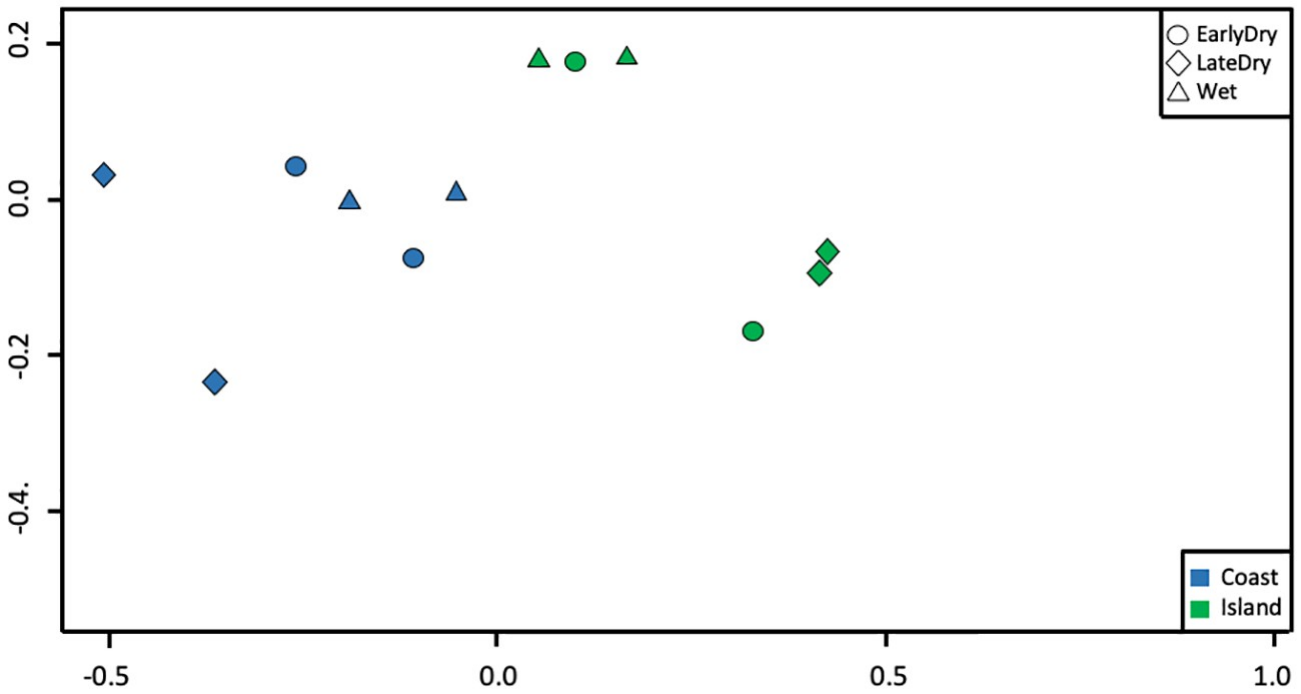
nMDS plot showing the grouping of sites and seasons. A clear separation of the islands site from the coast site was observed. Additionally, wet season sampling periods form a loose cluster separate from the dry seasons.

A SIMPER on 12 groups (all combinations of season, site, and year) indicated a significant difference in elasmobranch assemblage between the coast site and islands site (N permutations = 4,999, p = 0.007). Blacktip reef sharks contributed the most to the difference in assemblage between sites and were more abundant at the islands site, followed by the Bluespotted Maskrays, which were more abundant at the coast site (S3 Table). An nMDS plot also showed a divide between elasmobranch assemblage at the islands and at the coast (Fig 1). The groups from the wet season, although not tightly clustered, were also separated from the two dry season sampling periods.

### Temporal variations in abundance

**Fig 2:**
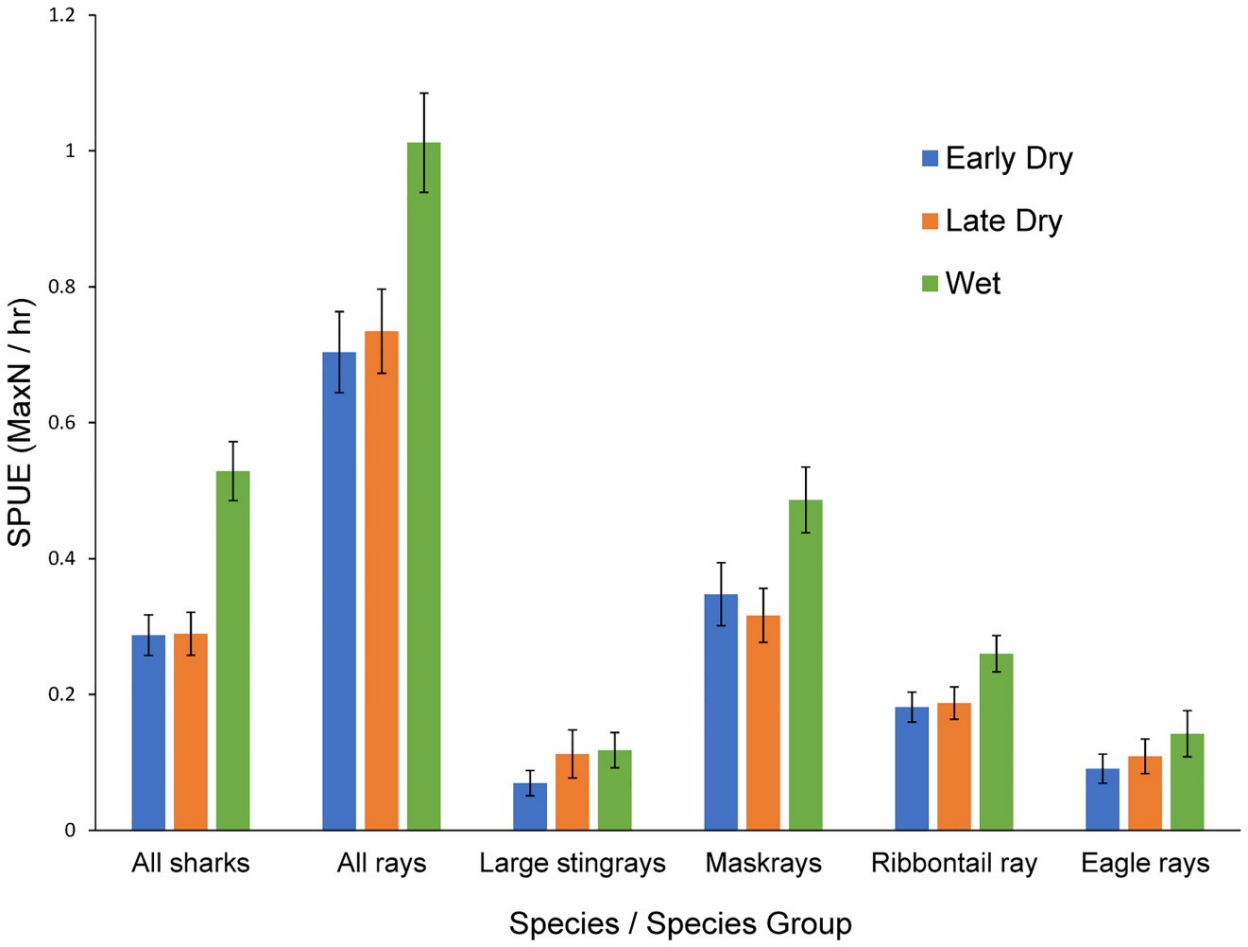
Sightings per unit effort (SPUE) of the six different species/ species groups analysed. All sharks, maskrays and bluespotted ribbontail rays had significantly higher abundances in the wet season than both the early and late dry season as per a PERMANOVA.

There were no significant differences in abundance for any species between the early dry and late dry seasons as shown by Tukey post-hoc tests of ANOVAs (all p>0.05) (Fig 2). As both early and late dry seasons provided the same abundances for each species/ species group, they were combined to a single ‘dry season’ for analysis of environmental factors affecting abundance.

ANOVAs showed that depths were not significantly different between sites or seasons (Sites: SS = 132, df = 1, 945, F = 1.4, p = 0.237; Season: SS = 76, df = 2, 944, F = 0.398, p = 0.671). Relief was not significantly different between seasons (SS = 1.0, df = 2, 944, F = 0.497, p = 0.609). The islands site had significantly higher relief as shown by an ANOVA (SS = 88.5, df = 1, 945, F = 96.43, p <0.001).

Generalised linear models (GLMs) showed season, relief, site, and depth to be significant factors contributing to rays observed and their abundances. Varying combinations of those factors were important to different species/ species groups (Table 2). Visibility was a contributing factor in the top model for eagle rays. Eagle rays were often observed incidentally in the distance, therefore, this inclusion in the top model was expected. For large stingrays, the top performing model was the null model (Table 2), indicating there was no evidence that environmental factors had anything other than random effects on the detection of this group of species when analysed together.

**Table 2:** Top GLM models for predicting SPUE (MaxN/hour) of the six species/species groups analysed.

Species Group	Model	ΔAIC	wAIC	Biggest VIF
All rays ‘ZINB’	**Season + Site + Relief + Depth**	**0**	**0.85**	**1.34**
	Season + Relief + Depth	3.54	0.15	1.18
	Relief + Depth + Site	14.82	0	1.34
	Null	103.12	0	-
Maskrays ‘ZINB’	**Season + Relief + Depth**	**0**	**0.50**	**1.79**
	Season + Relief + Site + Depth	0.33	0.43	1.34
	Season + Site*Relief	4.18	0.06	4.38
	Null	227.80	0	-
Ribbontail ray ‘poisson’	Site + Depth*Season	0	0.36	5.51
	**Season + Site + Depth**	**0.70**	**0.25**	**1.00**
	Site + Depth*Relief	1.09	0.21	4.40
	Null	65.13	0	-
Eagle rays ‘poisson’	**Site + Visibility**	**0**	**0.55**	**1.12**
	Site + Season + Visibility	1.95	0.21	1.36
	Site + Depth*Season	3.58	0.09	5.51
	Null	29.72	0	-
Large stingrays ‘ZINB’	**Null**	**0**	**1**	**-**
	Site*Relief + Season	23.09	0	4.38
	Site*Relief	24.62	0	4.38
	UnconsolidatedHabitat	30.35	0	-
All sharks ‘ZINB’	Season + Site*Relief	0	0.93	4.38
	**Season + Site + Relief**	**5.82**	**0.05**	**1.10**
	Season + Site + Relief + Depth	7.82	0.02	1.34
	Null	114.98	0	-

Difference between lowest corrected Akaike Information Criterion (ΔAICc), AIC weights (wAICc), and biggest VIF value of all variables in the model (Biggest VIF) are reported. Model selection was based on the most parsimonious model within two units of the lowest ΔAICc and with the biggest VIF value <3. Selected models are presented in bold. Variable codes: Relief—on a scale of 0–5 with increasing complexity, Season—wet or dry, Site—coast or islands, Depth—in meters, Visibility—water visibility in 2m bins (0–2, 2.1–4, 4.1–6, 6.1–8, 8.1–10, 10+).

‘ZINB’ indicates zero-inflated negative binomial distribution and ‘poisson’ indicates poisson distribution.

**Fig 3:**
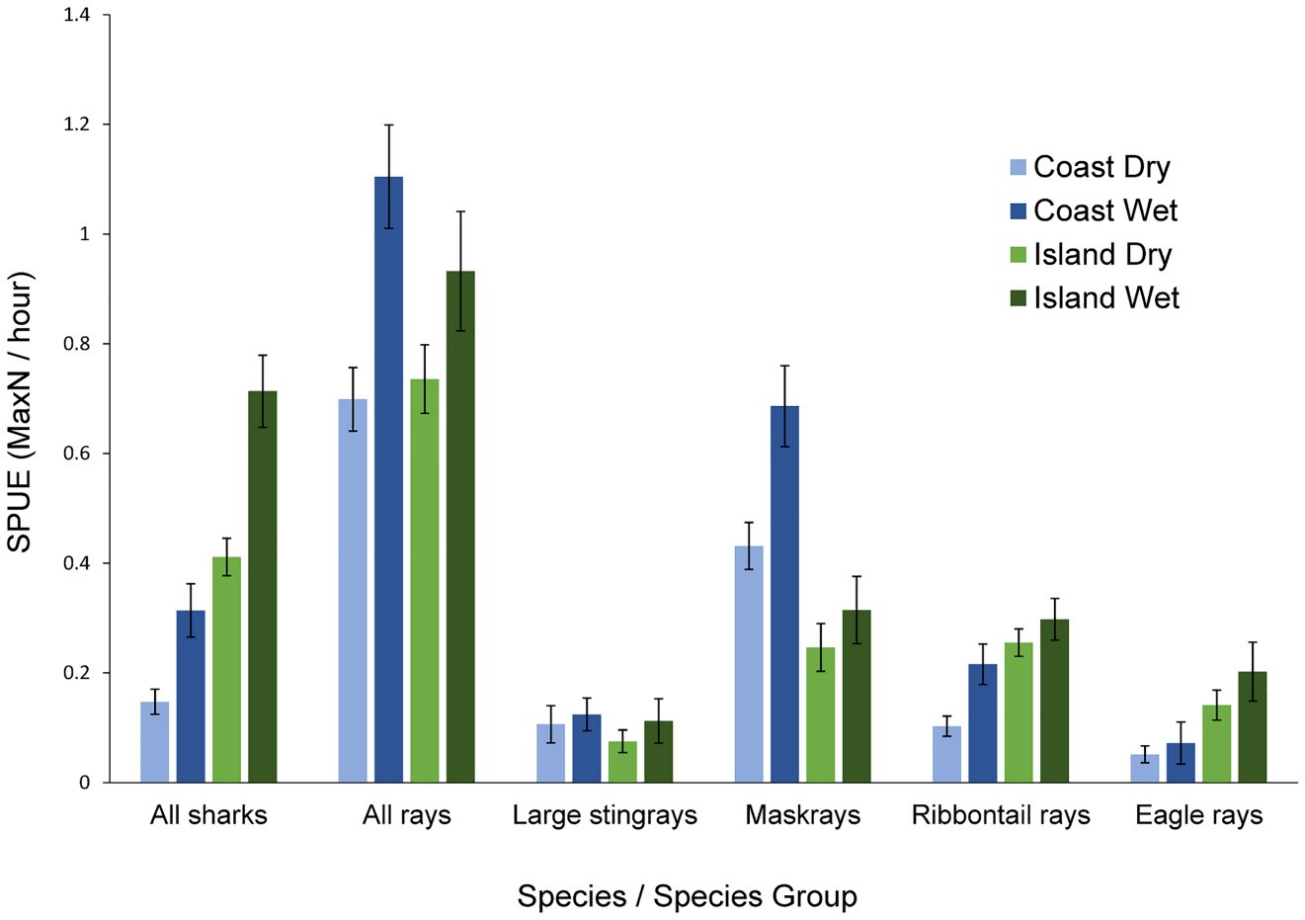
Sightings per unit effort (SPUE) of the six different species/ species groups analysed at the two sites in both seasons (early and late dry seasons are combined). Season was a significant influencing factor in abundance for all sharks, all rays, maskrays, and ribbontail rays will all four species/ species groups having higher abundances in the wet season.

**Fig 4:**
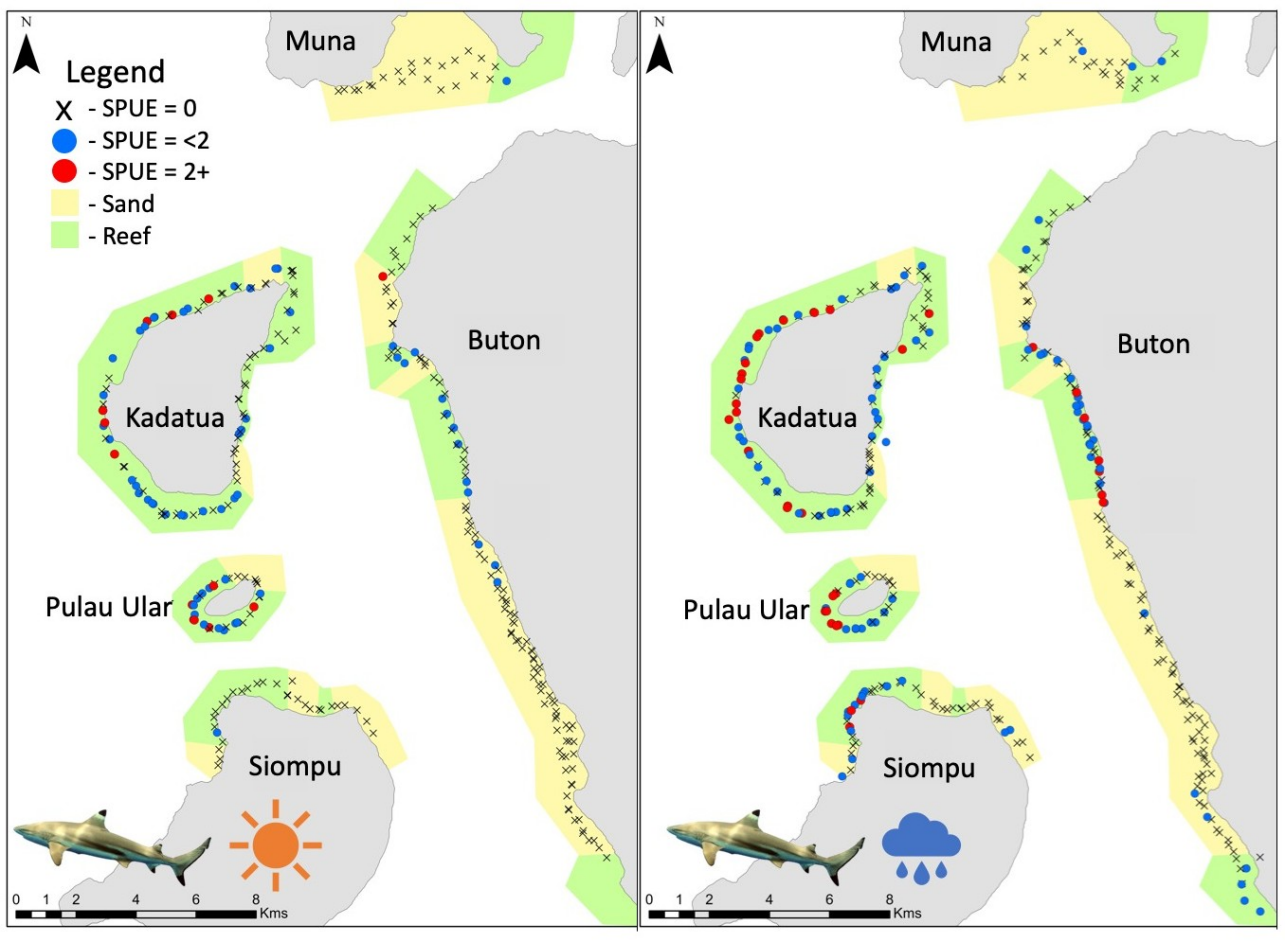
Abundance (SPUE) of sharks in Bau Bau, Sulawesi, Indonesia in the late dry (left), and wet (right) seasons. Sightings were significantly higher in the wet season than both dry seasons, which were not statistically different from one another. Higher concentrations of sharks were observed on the western side of each island, particularly in the wet season. Black Xs indicate BRUVS deployments with no sharks, blue circles indicate deployments with a single shark, while red dots indicate deployments with multiple sharks present.

**Fig 5:**
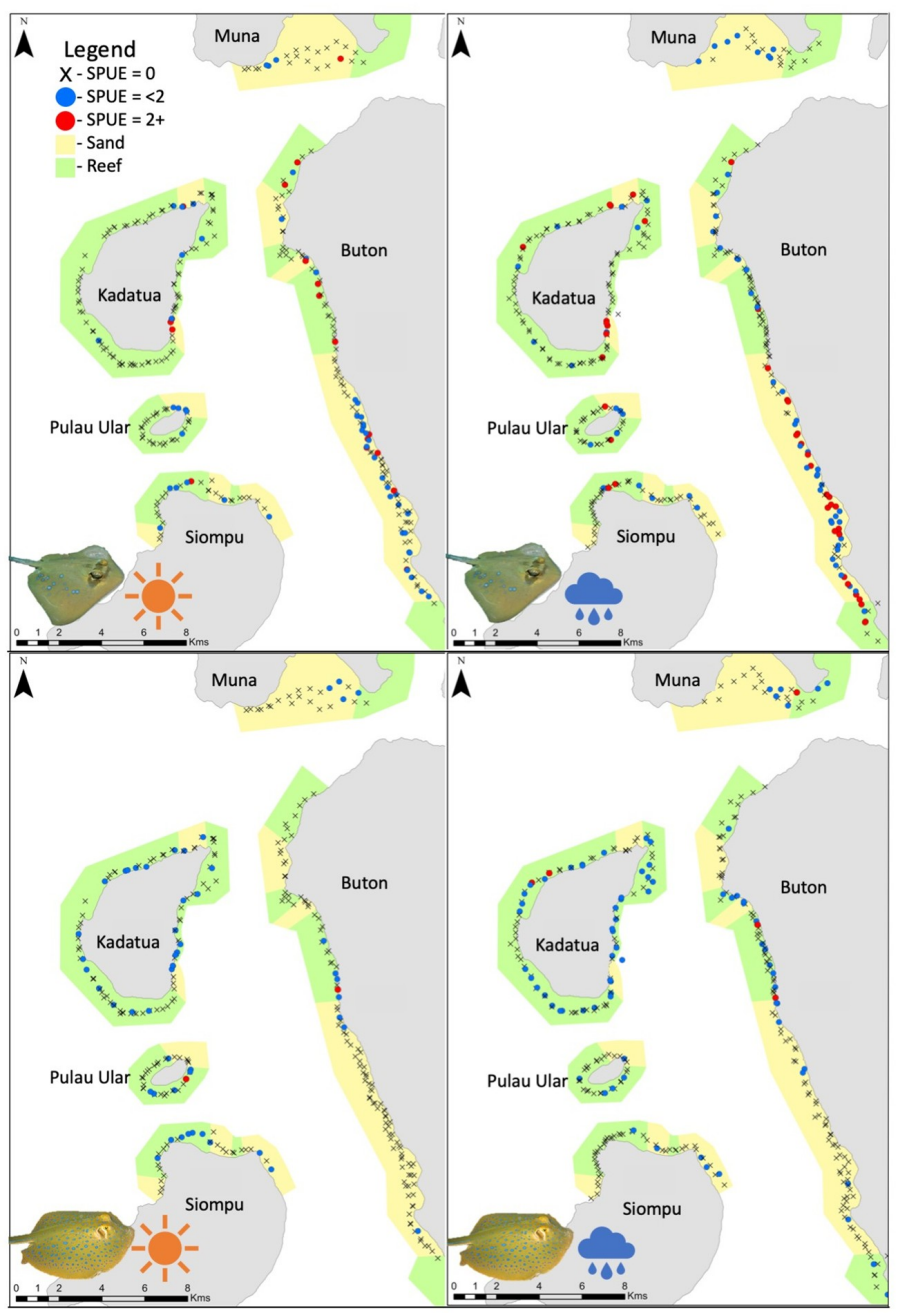
Abundance (SPUE) of the two most common ray groups in Bau Bau, Sulawesi, Indonesia. SPUE of bluespotted maskrays (*Neotrygon* spp.)(top) and bluespotted fantail rays (*Taeniura lymma*)(bottom) in the late dry (left) and wet (right) seasons. Sightings were significantly higher in the wet season than both dry seasons, which were not statistically different from one another. Higher abundances of maskrays were observed in sandy habitat (yellow) and higher abundances of ribbontail rays were observed at coral reef habitats (green). Black Xs indicate BRUVS deployments with no rays, blue circles indicate deployments with a single ray, while red dots indicate deployments with multiple rays present.

All sharks, all rays, maskrays, and ribbontail rays had higher SPUE values in the wet season than the dry season as shown by the inclusion of ‘season’ in the top GLM models (Figs 3–5). Site was also a significant factor for abundance of four species/ species groups (all sharks, all rays, ribbontail rays, and eagle rays). These groups, with the exception of ‘all rays’ were significantly higher in abundance at the islands site than the coast site (Figs 3–5).

**Fig 6:**
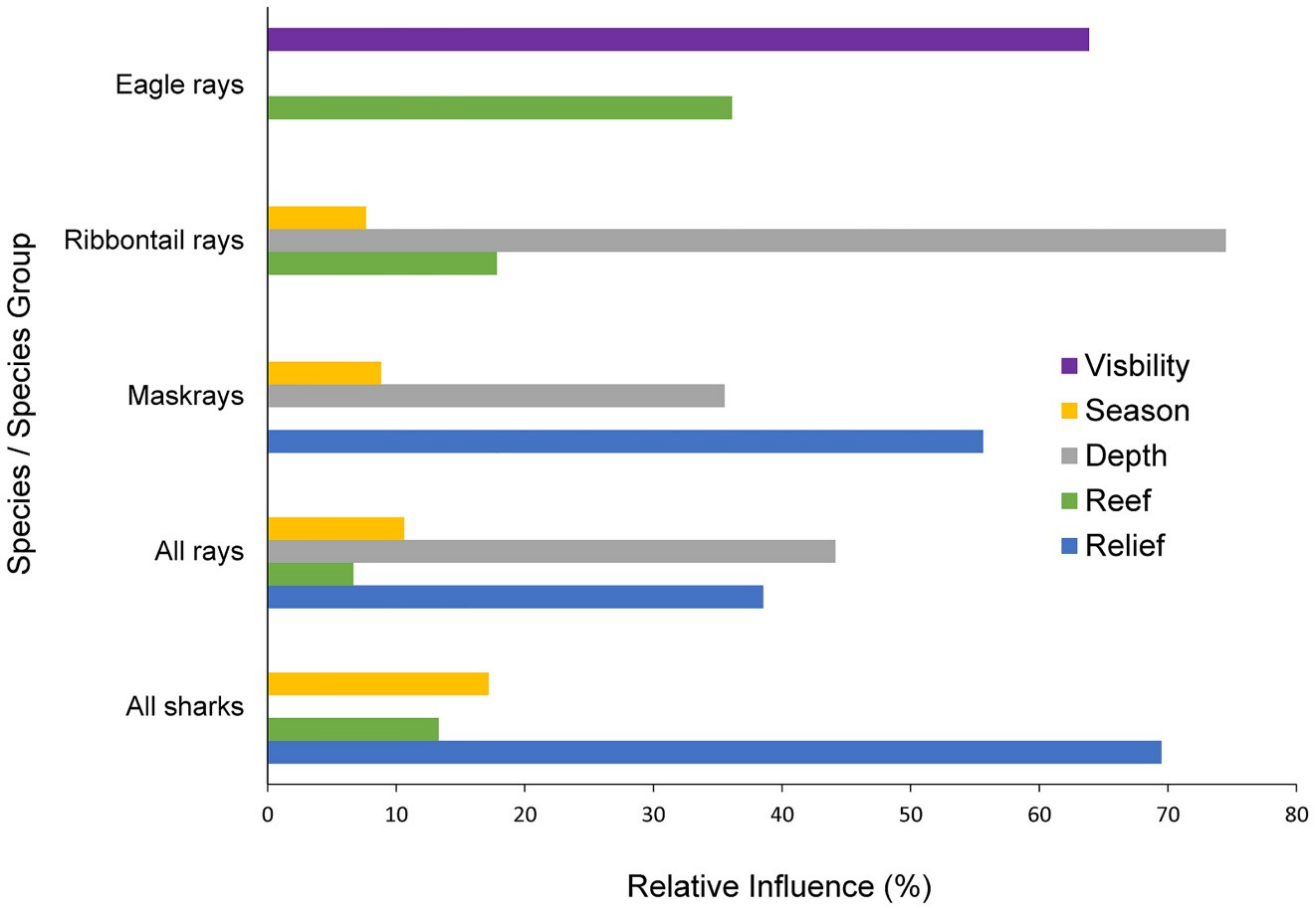
Relative influences of the explanatory variables for the different species / species groups based on generalized boosted regression models (GBM). Relief and depth were the two most important variables overall, while visibility was the most important variable in eagle ray abundance.

Generalised boosted regression models (GBMs) showed relative influence of relief was the greatest contributing variable for all sharks and maskray abundances (70% and 56%, respectively), and was also high for all rays (39%) (Fig 6). Depth was the greatest or second greatest contributing variable (over 35%) for all rays, ribbontail rays, and maskray abundances. Both site and season were contributing variables in four of the six species/ species groups. For eagle rays, visibility was the highest contributing variable at 64% (Fig 6).

Sharks were observed in higher abundances on the western side of each island and Buton in both the dry and wet seasons (Fig 4). The two ray genera that were most commonly observed had opposing habitat preferences [56]. Higher sightings of ribbontail rays were observed in areas with healthy coral reef habitat and higher abundances of maskrays were observed in sandy habitats, with little overlap of the two species (Fig 5). There are higher concentrations of coral reefs at the islands site associated with higher abundances of ribbontail rays at the islands than the coast. Similarly, the coast consists of a few reef patches and mostly sandy habitat leading to higher abundances of the maskrays (Fig 3).

## Discussion

The results of this study show that BRUVS are an appropriate survey method to capture spatial and temporal variation in elasmobranch abundances. We showed that BRUVS deployed in the same location, even when deployed within one month of initial sampling, can provide consistent results for both elasmobranch abundance and assemblage. Similar species composition and SPUEs of all species were observed within the same season across both years. However, BRUVS revealed distinct seasonal differences in shark and ray abundance on the coral reefs surrounding Bau Bau, Indonesia, and that this seasonal difference was consistent between years. Thus, while this study affirms that BRUVS are a reliable sampling method for abundance and elasmobranch assemblage comparisons, seasonal differences may need to be accounted for when comparing locations throughout the year or between years when sampling in different seasons. Elasmobranch species composition did not change significantly between seasons indicating a consistent composition of species. This result was expected as there are few migratory elasmobranch species in the area [57]. This also suggests that any seasonal differences observed were the result of changing abundances of each species within the elasmobranch community. However, the reason for these differences in abundance is unknown.

Seasonal differences in abundance seemingly contradict existing data that show blacktip reef sharks, bluespotted maskrays, and bluespotted ribbontail rays are highly reef associated and non-migratory species [58–60]. Blacktip reef sharks comprised 89% of sharks observed in this study and have not been observed to be seasonally resident in other regions of the world [61, 62]. Males on inshore reefs moved more and further distances during the reproductive season (Nov—Mar in Australia) [61], equivalent to the wet season in Bau Bau, which may explain their higher abundances at that time. Females have been documented to make movements of up to 50 km from their home range for parturition [63]. If females are moving for parturition and males are venturing further during the wet season, this may explain the reduced abundances. However, the change in abundance observed was high and likely not fully explained by these movements. Additionally, juvenile blacktip reef sharks have been observed in the area, suggesting the area contains viable habitat for juveniles and, therefore, pupping. The waters surrounding Bau Bau have high concentrations of mesophotic corals (Erika Gress, pers comms), meaning higher abundances of fish can be supported in deeper waters providing a food resource for sharks [64]. However, water temperatures are cooler in the dry season, thus it seems unlikely sharks would spend time in deeper, cooler water as previous studies have provided evidence of behavioural thermoregulation in this species [65]. Further research is needed to determine the movement and behavioural patterns of reef sharks surrounding Bau Bau to determine if the observed trend persists over a longer time frame and additional environmental variables should be tested across seasons to gain a better understanding of why their abundances differ on BRUVS between seasons.

Bluespotted maskrays comprised 47% of all rays observed and although no data on their movement patterns exist, one study captured individuals after 3 years of liberty within 40 km of where they were tagged, suggesting relatively small home ranges [59]. These rays were 128–159% more abundant in the wet season at the coast and islands site, respectively, which likely has significant ecological influence. There is no distinct breeding season in maskrays and in a captive population, mating occurred soon after parturition [66]. Therefore, movement for mating seems unlikely and would not explain the patterns observed here. These rays occur in high abundances in Southeast Asia in sandy habitats adjacent to reefs where they can feed on benthic, sand-dwelling invertebrates [67]. The second most abundant species of ray, the bluespotted ribbontail ray, comprised 26% of rays observed. Bluespotted ribbontail rays were also more abundant in the wet season than dry with abundances 116–210% higher at the coast and islands sites, respectively. These rays are extremely dependent on coral reefs as they use corals for protection while resting, meaning movement away from their reef is unlikely between seasons [60].

Abiotic factors may play a large role in the ability to observe elasmobranchs on BRUVS between the different seasons. In the wet season, the prevailing wind is from the west, when higher abundances of sharks were observed. Wind speed and duration can greatly affect the nutrient load within a region [68]. Despite the wind changes, visibility was not significantly different between seasons, therefore, this was not a contributing factor in the different abundances observed. While the wind patterns may provide some explanation for why sharks were observed on the western side of the islands, individuals do not appear to shift to the east side of the smaller islands in the dry season when winds change and come from the southeast. Reef sharks are able to travel further than rays, so it is possible that a portion of the population seasonally migrates to the east side of Buton, which was not sampled in this study. However, if this is occurring, the drivers of these movements are not known.

Although the area has a relatively stable temperature throughout the year, nutrient loads may still be affected by winds and other environmental factors during different seasons [69]. South Sulawesi has a noted peak in chlorophyll *a* around July, during the dry season sampling period [40]. Dissolved oxygen levels can dramatically shift diurnally in these eutrophic periods with high oxygen levels during daylight hours when photosynthesis is occurring and low oxygen levels at night [70]. Changes in oxygen levels may affect shark and ray movement, and therefore, their detectability on BRUVS. With lower oxygen levels at night in the dry season, elasmobranch activity may be reduced during the day. For example, in the bonnethead shark (*Sphyrna tiburo*) lower dissolved oxygen levels led to increased swimming and higher activity rates [3]. There may be fewer individuals observed in the dry season as they move more at night, to account for the lower oxygen levels. Oxygen consumption in elasmobranchs has been shown to increase with increasing temperature [71], therefore, sharks and rays may have higher activity rates in the warmer months (wet season) due to increased oxygen consumption. Additionally, as ectotherms, elasmobranchs may be more active due to the increase in temperature [72]. With higher activity levels (movement), there would be a higher likelihood of encountering a bait plume and following it to the BRUVS. Ribbontail rays have higher metabolic performance in warmer water temperatures [73]. Similarly, juvenile blacktip reef sharks have high physiological performances in warmer, shallow waters [74], thus, this may explain both these species’ apparent increased abundances in the wet season.

Time of day was not a significant factor in the presence of any elasmobranch species as it was not included in any of the top GLM models, however, sampling in this study only occurred during daylight hours. Some elasmobranch species, like whitetip reef sharks are nocturnal feeders so may not be attracted to bait set during daylight hours [75]. Few whitetip reef sharks were observed in this study, potentially because they were not actively hunting during BRUVS deployment times [50]. Stingray diel patterns are not well understood and there are apparent species-specific differences in total activity diurnally, with some more active at night and others consistently active throughout the day [76, 77]. No movement information on the ray species observed in this study was available.

There were significantly higher abundances of sharks and rays at the islands site, which was likely the result of higher fishing pressure at the coast site. Sharks have been shown to be more abundant in areas with lower human populations [78]. The coast has a much higher human population than any of the three islands, two of which are inhabited with a few small villages. The primary fishing vessels used in the Bau Bau region are small dugout canoes, sometimes with a small motor (Sherman, pers. obs). These canoes are not powerful enough to travel from the coast to the islands for fishing. Therefore, only larger boats and island locals are able to fish at the islands site. Additionally, due to the large population in Bau Bau, the coast is subject to high levels of contamination from sewage, rubbish, noise, and other pollutants that may affect elasmobranch abundances [79, 80]. These pollutants may also affect the habitat quality, therefore, the island site likely has preferable habitat. Although not remote, this shows that ease of access to fishing grounds and other anthropogenic factors can impact species composition and abundance [81].

In conclusion, the results from this study demonstrate that while BRUVS provide a reliable and repeatable method for surveying elasmobranchs, care must be taken in timing of sampling across different regions to ensure valid and legitimate comparisons between multiple locations. Although the site sampled was tropical with minimal seasonal changes in temperature and weather conditions, there were significantly different abundances of both sharks and rays across seasons meaning studies comparing sites should be performed in the same season to achieve accurate comparisons. In large scale studies that span several countries (e.g. [82]), it is not always feasible to complete surveys in the same season. Our results demonstrate that the elasmobranch assemblage did not differ between seasons, thus any conclusions made regarding species composition should be accurate. This is expected as coral reef species tend to be present year-round. Timing of sampling should, however, be considered in analyses and conclusions. Further investigation analysing invertebrate and fish biomass, dissolved oxygen, and other environmental variables should be done to determine if some other factor may be influencing elasmobranch presence or detectability throughout the year.

## Supporting information

S1 Table List of possible species within maskrays, eagle rays, and devil/manta rays. Possible species based on geographic range and similar appearance within the three groups.
(DOCX)

S2 Table List of all models tested for each species/species group. Models were run using the glmmTMB package in R with a zero inflation of 1.
(DOCX)

S3 Table Results of SIMPER analysis to determine the impact of each species/species group to elasmobranch community composition differences between sites. (DOCX)
