# Supplementary material for: Repeatability of baited remote underwater video station (BRUVS) results within and between seasons
Source: PLoS One. 2020 Dec 17;15(12):e0244154. doi: 10.1371/journal.pone.0244154 (PMC7745976; doi:10.1371/journal.pone.0244154)
Supplement: S1 Table — Possible species based on geographic range and similar appearance within the three groups. (DOCX) [file pone.0244154.s001.docx]

**S1 Table.** List of Possible Species Within Maskrays, Eagle Rays, and Devil/Manta Rays. Possible species based on geographic range and similar appearance within the three groups.

| Species Group | Common Name | Latin Name | Species Authority |
| --- | --- | --- | --- |
| Maskrays (*Neotrygon*) | Plain maskray | *Neotrygon annotata* | Last, 1987 |
|  | Australian bluespotted maskray | *Neotrygon australiae* | Last, White and Séret, 2016 |
|  | Bluespotted maskray | *Neotrygon caeruleopunctata* | Last, White and Séret, 2016 |
|  | Oriental bluespotted maskray | *Neotrygon orientalis* | Last, White and Séret, 2016 |
| Eagle rays (*Aetobatus / Aetomylaeus*) | Mottled eagle ray | *Aetomylaeus maculatus* | Gray, 1834 |
|  | Banded eagle ray | *Aetomylaeus nichofii* | Bloch and Schneider, 1801 |
|  | Ornate eagle ray | *Aetomylaeus vespertilio* | Bleerker, 1852 |
|  | Longhead eagle ray | *Aetobatus flagellum* | Bloch and Schneider, 1801 |
|  | Spotted eagle ray | *Aetobatus ocellatus* | Kuhl, 1823 |
| Devil / Manta rays (*Mobula*) | Reef manta ray | *Mobula alfredi* | Krefft, 1868 |
|  | Giant manta ray | *Mobula birostris* | Walbaum, 1792 |
|  | Kuhl’s devilray | *Mobula kuhlii* | Müller and Henle, 1841 |
|  | Giant devilray | *Mobula mobular* | Bonnaterre, 1788 |
|  | Chilean devilray | *Mobula tarapacana* | Philippi, 1892 |
|  | Bentfin devilray | *Mobula thurstoni* | Lloyd, 1908 |
