# Supplementary material for: Repeatability of baited remote underwater video station (BRUVS) results within and between seasons
Source: PLoS One. 2020 Dec 17;15(12):e0244154. doi: 10.1371/journal.pone.0244154 (PMC7745976; doi:10.1371/journal.pone.0244154)
Supplement: S2 Table — Models were run using the glmmTMB package in R with a zero inflation of 1. (DOCX) [file pone.0244154.s002.docx]

**S2 Table.** List of all models tested for each species / species group. Models were run using the glmmTMB package in R with a zero inflation of 1.

| **Species** | **Model Number** | **Variables Included** | **Distribution** |
| --- | --- | --- | --- |
| All sharks | 1 | Null | Negative binomial |
| All sharks | 2 | Site | Negative binomial |
| All sharks | 3 | Season | Negative binomial |
| All sharks | 4 | Trip* | Negative binomial |
| All sharks | 5 | Reef habitat (flat, crest, or slope) | Negative binomial |
| All sharks | 6 | Year | Negative binomial |
| All sharks | 7 | Depth | Negative binomial |
| All sharks | 8 | Visibility | Negative binomial |
| All sharks | 9 | Relief | Negative binomial |
| All sharks | 10 | Unconsolidated habitat (% cover) | Negative binomial |
| All sharks | 11 | Hard coral (% cover) | Negative binomial |
| All sharks | 12 | Time of day (morning, midday, or afternoon) | Negative binomial |
| All sharks | 13 | Tide (ebb, slack, or flow) | Negative binomial |
| All sharks | 14 | Wind speed (Beaufort scale) | Negative binomial |
| All sharks | 15 | Cloud cover (%) | Negative binomial |
| All sharks | 16 | Wind direction | Negative binomial |
| All sharks | 17 | Surface conditions (low, medium, or high wave action) | Negative binomial |
| All sharks | 18 | Site + Season | Negative binomial |
| All sharks | 19 | Site * Season | Negative binomial |
| All sharks | 20 | Site + Relief | Negative binomial |
| All sharks | 21 | Site * Relief | Negative binomial |
| All sharks | 22 | Site + Relief + Season | Negative binomial |
| All sharks | 23 | Site * Relief + Season | Negative binomial |
| All sharks | 24 | Site + Relief * Season | Negative binomial |
| All sharks | 25 | Site * Relief + Season + Trip | Negative binomial |
| All sharks | 26 | Site + Depth | Negative binomial |
| All sharks | 27 | Site + Depth + Relief + Season | Negative binomial |
| All sharks | 28 | Site + Relief * Season + Trip | Negative binomial |
| All sharks | 29 | Site + Depth * Relief | Negative binomial |
| All sharks | 30 | Site + Depth * Season | Negative binomial |
| All sharks | 31 | Site + Season + Visibility | Negative binomial |
| All sharks | 32 | Site + Depth + Relief | Negative binomial |
| All sharks | 33 | Site + Depth + Season | Negative binomial |
| All sharks | 34 | Depth + Season + Relief | Negative binomial |
| All sharks | 35 | SPUE of All Rays | Negative binomial |
| All rays | 1 | Null | Negative binomial |
| All rays | 2 | Site | Negative binomial |
| All rays | 3 | Season | Negative binomial |
| All rays | 4 | Trip* | Negative binomial |
| All rays | 5 | Reef habitat (flat, crest, or slope) | Negative binomial |
| All rays | 6 | Year | Negative binomial |
| All rays | 7 | Depth | Negative binomial |
| All rays | 8 | Visibility | Negative binomial |
| All rays | 9 | Relief | Negative binomial |
| All rays | 10 | Unconsolidated habitat (% cover) | Negative binomial |
| All rays | 11 | Hard coral (% cover) | Negative binomial |
| All rays | 12 | Time of day (morning, midday, or afternoon) | Negative binomial |
| All rays | 13 | Tide (ebb, slack, or flow) | Negative binomial |
| All rays | 14 | Wind speed (Beaufort scale) | Negative binomial |
| All rays | 15 | Cloud cover (%) | Negative binomial |
| All rays | 16 | Wind direction | Negative binomial |
| All rays | 17 | Surface conditions (low, medium, or high wave action) | Negative binomial |
| All rays | 18 | Site + Season | Negative binomial |
| All rays | 19 | Site * Season | Negative binomial |
| All rays | 20 | Site + Relief | Negative binomial |
| All rays | 21 | Site * Relief | Negative binomial |
| All rays | 22 | Site + Relief + Season | Negative binomial |
| All rays | 23 | Site * Relief + Season | Negative binomial |
| All rays | 24 | Site + Relief * Season | Negative binomial |
| All rays | 25 | Site * Relief + Season + Trip | Negative binomial |
| All rays | 26 | Site + Depth | Negative binomial |
| All rays | 27 | Site + Depth + Relief + Season | Negative binomial |
| All rays | 28 | Site + Relief * Season + Trip | Negative binomial |
| All rays | 29 | Site + Depth * Relief | Negative binomial |
| All rays | 30 | Site + Depth * Season | Negative binomial |
| All rays | 31 | Site + Season + Visibility | Negative binomial |
| All rays | 32 | Site + Depth + Relief | Negative binomial |
| All rays | 33 | Site + Depth + Season | Negative binomial |
| All rays | 34 | Depth + Season + Relief | Negative binomial |
| All rays | 35 | SPUE of All Sharks | Negative binomial |
| Maskrays | 1 | Null | Negative binomial |
| Maskrays | 2 | Site | Negative binomial |
| Maskrays | 3 | Season | Negative binomial |
| Maskrays | 4 | Trip* | Negative binomial |
| Maskrays | 5 | Reef habitat (flat, crest, or slope) | Negative binomial |
| Maskrays | 6 | Year | Negative binomial |
| Maskrays | 7 | Depth | Negative binomial |
| Maskrays | 8 | Visibility | Negative binomial |
| Maskrays | 9 | Relief | Negative binomial |
| Maskrays | 10 | Unconsolidated habitat (% cover) | Negative binomial |
| Maskrays | 11 | Hard coral (% cover) | Negative binomial |
| Maskrays | 12 | Time of day (morning, midday, or afternoon) | Negative binomial |
| Maskrays | 13 | Tide (ebb, slack, or flow) | Negative binomial |
| Maskrays | 14 | Wind speed (Beaufort scale) | Negative binomial |
| Maskrays | 15 | Cloud cover (%) | Negative binomial |
| Maskrays | 16 | Wind direction | Negative binomial |
| Maskrays | 17 | Surface conditions (low, medium, or high wave action) | Negative binomial |
| Maskrays | 18 | Site + Season | Negative binomial |
| Maskrays | 19 | Site * Season | Negative binomial |
| Maskrays | 20 | Site + Relief | Negative binomial |
| Maskrays | 21 | Site * Relief | Negative binomial |
| Maskrays | 22 | Site + Relief + Season | Negative binomial |
| Maskrays | 23 | Site * Relief + Season | Negative binomial |
| Maskrays | 24 | Site + Relief * Season | Negative binomial |
| Maskrays | 25 | Site * Relief + Season + Trip | Negative binomial |
| Maskrays | 26 | Site + Depth | Negative binomial |
| Maskrays | 27 | Site + Depth + Relief + Season | Negative binomial |
| Maskrays | 28 | Site + Relief * Season + Trip | Negative binomial |
| Maskrays | 29 | Site + Depth * Relief | Negative binomial |
| Maskrays | 30 | Site + Depth * Season | Negative binomial |
| Maskrays | 31 | Site + Season + Visibility | Negative binomial |
| Maskrays | 32 | Site + Depth + Relief | Negative binomial |
| Maskrays | 33 | Site + Depth + Season | Negative binomial |
| Maskrays | 34 | Depth + Season + Relief | Negative binomial |
| Maskrays | 35 | SPUE of All Sharks | Negative binomial |
| Ribbontail rays | 1 | Null | Poisson |
| Ribbontail rays | 2 | Site | Poisson |
| Ribbontail rays | 3 | Season | Poisson |
| Ribbontail rays | 4 | Trip* | Poisson |
| Ribbontail rays | 5 | Reef habitat (flat, crest, or slope) | Poisson |
| Ribbontail rays | 6 | Year | Poisson |
| Ribbontail rays | 7 | Depth | Poisson |
| Ribbontail rays | 8 | Visibility | Poisson |
| Ribbontail rays | 9 | Relief | Poisson |
| Ribbontail rays | 10 | Unconsolidated habitat (% cover) | Poisson |
| Ribbontail rays | 11 | Hard coral (% cover) | Poisson |
| Ribbontail rays | 12 | Time of day (morning, midday, or afternoon) | Poisson |
| Ribbontail rays | 13 | Tide (ebb, slack, or flow) | Poisson |
| Ribbontail rays | 14 | Wind speed (Beaufort scale) | Poisson |
| Ribbontail rays | 15 | Cloud cover (%) | Poisson |
| Ribbontail rays | 16 | Wind direction | Poisson |
| Ribbontail rays | 17 | Surface conditions (low, medium, or high wave action) | Poisson |
| Ribbontail rays | 18 | Site + Season | Poisson |
| Ribbontail rays | 19 | Site * Season | Poisson |
| Ribbontail rays | 20 | Site + Relief | Poisson |
| Ribbontail rays | 21 | Site * Relief | Poisson |
| Ribbontail rays | 22 | Site + Relief + Season | Poisson |
| Ribbontail rays | 23 | Site * Relief + Season | Poisson |
| Ribbontail rays | 24 | Site + Relief * Season | Poisson |
| Ribbontail rays | 25 | Site * Relief + Season + Trip | Poisson |
| Ribbontail rays | 26 | Site + Depth | Poisson |
| Ribbontail rays | 27 | Site + Depth + Relief + Season | Poisson |
| Ribbontail rays | 28 | Site + Relief * Season + Trip | Poisson |
| Ribbontail rays | 29 | Site + Depth * Relief | Poisson |
| Ribbontail rays | 30 | Site + Depth * Season | Poisson |
| Ribbontail rays | 31 | Site + Season + Visibility | Poisson |
| Ribbontail rays | 32 | Site + Depth + Relief | Poisson |
| Ribbontail rays | 33 | Site + Depth + Season | Poisson |
| Ribbontail rays | 34 | Depth + Season + Relief | Poisson |
| Ribbontail rays | 35 | SPUE of All Sharks | Poisson |
| Eagle rays | 1 | Null | Poisson |
| Eagle rays | 2 | Site | Poisson |
| Eagle rays | 3 | Season | Poisson |
| Eagle rays | 4 | Trip* | Poisson |
| Eagle rays | 5 | Reef habitat (flat, crest, or slope) | Poisson |
| Eagle rays | 6 | Year | Poisson |
| Eagle rays | 7 | Depth | Poisson |
| Eagle rays | 8 | Visibility | Poisson |
| Eagle rays | 9 | Relief | Poisson |
| Eagle rays | 10 | Unconsolidated habitat (% cover) | Poisson |
| Eagle rays | 11 | Hard coral (% cover) | Poisson |
| Eagle rays | 12 | Time of day (morning, midday, or afternoon) | Poisson |
| Eagle rays | 13 | Tide (ebb, slack, or flow) | Poisson |
| Eagle rays | 14 | Wind speed (Beaufort scale) | Poisson |
| Eagle rays | 15 | Cloud cover (%) | Poisson |
| Eagle rays | 16 | Wind direction | Poisson |
| Eagle rays | 17 | Surface conditions (low, medium, or high wave action) | Poisson |
| Eagle rays | 18 | Site + Season | Poisson |
| Eagle rays | 19 | Site * Season | Poisson |
| Eagle rays | 20 | Site + Relief | Poisson |
| Eagle rays | 21 | Site * Relief | Poisson |
| Eagle rays | 22 | Site + Relief + Season | Poisson |
| Eagle rays | 23 | Site * Relief + Season | Poisson |
| Eagle rays | 24 | Site + Relief * Season | Poisson |
| Eagle rays | 25 | Site * Relief + Season + Trip | Poisson |
| Eagle rays | 26 | Site + Depth | Poisson |
| Eagle rays | 27 | Site + Depth + Relief + Season | Poisson |
| Eagle rays | 28 | Site + Relief * Season + Trip | Poisson |
| Eagle rays | 29 | Site + Depth * Relief | Poisson |
| Eagle rays | 30 | Site + Depth * Season | Poisson |
| Eagle rays | 31 | Site + Season + Visibility | Poisson |
| Eagle rays | 32 | Site + Depth + Relief | Poisson |
| Eagle rays | 33 | Site + Depth + Season | Poisson |
| Eagle rays | 34 | Depth + Season + Relief | Poisson |
| Eagle rays | 35 | SPUE of All Sharks | Poisson |
| Large stingrays | 1 | Null | Negative binomial |
| Large stingrays | 2 | Site | Negative binomial |
| Large stingrays | 3 | Season | Negative binomial |
| Large stingrays | 4 | Trip* | Negative binomial |
| Large stingrays | 5 | Reef habitat (flat, crest, or slope) | Negative binomial |
| Large stingrays | 6 | Year | Negative binomial |
| Large stingrays | 7 | Depth | Negative binomial |
| Large stingrays | 8 | Visibility | Negative binomial |
| Large stingrays | 9 | Relief | Negative binomial |
| Large stingrays | 10 | Unconsolidated habitat (% cover) | Negative binomial |
| Large stingrays | 11 | Hard coral (% cover) | Negative binomial |
| Large stingrays | 12 | Time of day (morning, midday, or afternoon) | Negative binomial |
| Large stingrays | 13 | Tide (ebb, slack, or flow) | Negative binomial |
| Large stingrays | 14 | Wind speed (Beaufort scale) | Negative binomial |
| Large stingrays | 15 | Cloud cover (%) | Negative binomial |
| Large stingrays | 16 | Wind direction | Negative binomial |
| Large stingrays | 17 | Surface conditions (low, medium, or high wave action) | Negative binomial |
| Large stingrays | 18 | Site + Season | Negative binomial |
| Large stingrays | 19 | Site * Season | Negative binomial |
| Large stingrays | 20 | Site + Relief | Negative binomial |
| Large stingrays | 21 | Site * Relief | Negative binomial |
| Large stingrays | 22 | Site + Relief + Season | Negative binomial |
| Large stingrays | 23 | Site * Relief + Season | Negative binomial |
| Large stingrays | 24 | Site + Relief * Season | Negative binomial |
| Large stingrays | 25 | Site * Relief + Season + Trip | Negative binomial |
| Large stingrays | 26 | Site + Depth | Negative binomial |
| Large stingrays | 27 | Site + Depth + Relief + Season | Negative binomial |
| Large stingrays | 28 | Site + Relief * Season + Trip | Negative binomial |
| Large stingrays | 29 | Site + Depth * Relief | Negative binomial |
| Large stingrays | 30 | Site + Depth * Season | Negative binomial |
| Large stingrays | 31 | Site + Season + Visibility | Negative binomial |
| Large stingrays | 32 | Site + Depth + Relief | Negative binomial |
| Large stingrays | 33 | Site + Depth + Season | Negative binomial |
| Large stingrays | 34 | Depth + Season + Relief | Negative binomial |
| Large stingrays | 35 | SPUE of All Sharks | Negative binomial |

*trip refers to the six sampling periods
