# Supplementary material for: Repeatability of baited remote underwater video station (BRUVS) results within and between seasons
Source: PLoS One. 2020 Dec 17;15(12):e0244154. doi: 10.1371/journal.pone.0244154 (PMC7745976; doi:10.1371/journal.pone.0244154)
Supplement: S3 Table — (DOCX) [file pone.0244154.s003.docx]

**S3 Table.** Results of SIMPER analysis to determine the impact of each species/species group to elasmobranch community composition differences between sites.

| Species | Average abundance | Sim/SD | Ratio | Contribution (%) | Cumulative (%) |
| --- | --- | --- | --- | --- | --- |
| Blacktip reef sharks | 0.129 | 0.075 | 1.717 | 33.05 | 33.05 |
| Maskrays | 0.090 | 0.057 | 1.583 | 22.97 | 56.02 |
| Ribbontail rays | 0.070 | 0.033 | 2.105 | 17.91 | 73.93 |
| Eagle rays | 0.054 | 0.031 | 1.762 | 13.89 | 87.82 |
| Whitetip reef sharks | 0.027 | 0.014 | 1.879 | 6.91 | 94.73 |
| Large stingrays | 0.018 | 0.015 | 1.181 | 4.50 | 99.23 |
| Devil rays | 0.003 | 0.003 | 0.971 | 0.77 | 100.00 |
